# Inhibition of IL-6 in the LCWE Mouse Model of Kawasaki Disease Inhibits Acute Phase Reactant Serum Amyloid A but Fails to Attenuate Vasculitis

**DOI:** 10.3389/fimmu.2021.630196

**Published:** 2021-04-09

**Authors:** Rebecca A. Porritt, Carol Chase Huizar, Edward J. Dick, Shyamesh Kumar, Renee Escalona, Angela C. Gomez, Stefani Marek-Iannucci, Magali Noval Rivas, Jean Patterson, Thomas G. Forsthuber, Moshe Arditi, Mark Gorelik

**Affiliations:** ^1^ Departments of Pediatrics, Division of Pediatric Infectious Diseases and Immunology, Cedars-Sinai Medical Center, Los Angeles, CA, United States; ^2^ Biomedical Sciences, Infectious and Immunologic Diseases Research Center, Cedars-Sinai Medical Center, Los Angeles, CA, United States; ^3^ Department of Biology, University of Texas San Antonio, San Antonio, TX, United States; ^4^ Southwest National Primate Research Center, Texas Biomedical Research Institute, San Antonio, TX, United States; ^5^ Texas Biomedical Research Institute, San Antonio, TX, United States; ^6^ Department of Pediatric Allergy, Immunology and Rheumatology, Columbia University Medical Center, New York, NY, United States

**Keywords:** Kawasaki disease, vasculitis, LCWE, magnetic and microwave proteomics, IL-6, STAT3, anakinra, IL-1

## Abstract

**Objective:**

Kawasaki disease (KD) is the most common cause of acquired pediatric heart disease in the developed world. 10% of KD patients are resistant to front-line therapy, and no interventions exist to address secondary complications such as myocardial fibrosis. We sought to identify proteins and pathways associated with disease and anti-IL-1 treatment in a mouse model of KD.

**Methods:**

Vasculitis was induced *via* Lactobacillus casei cell wall extract (LCWE) injection in 5-week-old male mice. Groups of mice were injected with LCWE alone, LCWE and IL-1 receptor antagonist anakinra, or saline for controls. Upper heart tissue was assessed by quantitative mass spectrometry analysis. Expression and activation of STAT3 was assessed by immunohistochemistry, immunofluorescence and Western blot, and IL-6 expression by RNA-seq and ELISA. A STAT3 small molecular inhibitor and anti-IL-6R antibody were used to evaluate the role of STAT3 and IL-6 in disease development.

**Results:**

STAT3 was highly expressed and phosphorylated in cardiac tissue of LCWE-injected mice, and reduced following anakinra treatment. *Il6* and *Stat*3 gene expression was enhanced in abdominal aorta of LCWE-injected mice and reduced with Anakinra treatment. IL-6 serum levels were enhanced in LCWE-injected mice and normalized by anakinra. However, neither inhibition of STAT3 nor blockade of IL-6 altered disease development.

**Conclusion:**

Proteomic analysis of cardiac tissues demonstrates differential protein expression between KD-like, control and anakinra treated cardiac tissue. STAT3 and IL-6 were highly upregulated with LCWE and normalized by anakinra treatment. However, both STAT3 and IL-6 were dispensable for disease development indicating they may be bystanders of inflammation.

## Introduction

Kawasaki disease (KD) is a relatively common febrile disease and vasculitis of childhood (10-20/100,000 incidence in North America) of unknown etiology, and typically presents as a febrile illness with features including rash, mucositis and conjunctivitis in children from ages 6 months to 5 years (1). While the acute process is self-limited, many patients with KD subsequently develop inflammatory vasculitic lesions of the coronary vasculature, resulting in coronary artery aneurysms (CAA). An emerging concern in the KD field is the recognition some patients develop late-onset myocardial fibrosis, which can lead to heart failure and death (1, 2).

Current management of KD is focused on preventing coronary artery aneurysms, and is anchored by the use of intravenous immunoglobulin (IVIG). IVIG suppresses the inflammatory response and reduces the number of patients afflicted with persistent CAA from 20% to around 5% if administered within the first 10 days of illness (3, 4). However, beyond surveillance for sequelae, there is no current management or therapy for myocardial fibrosis in KD (5). Despite current treatment and practices, KD remains the most common cause of acquired heart disease in children in the US and developed world (1). In addition, approximately 10% of all KD patients are resistant to initial IVIG therapy, and require adjunctive treatment (6).

While there is some understanding of the immunologic interplay that occurs in the acute phase of KD (7), many of the molecular pathological mechanisms that result in acute cardiac inflammation remain unclear. The current general consensus is that an unknown infectious agent promotes both innate and adaptive immunologic responses, resulting in an autoimmune attack on cardiac tissue (7). Co-localization of T cells and dendritic cells in and around lesions supports this hypothesis (7). Histopathological studies of autopsy specimens demonstrate infiltration of the vascular wall and adventitia by cells believed to be myofibroblasts or myofibroblast-like cells (8, 9). These cells may be responsible for the remodeling that results in coronary aneurysms and cardiac fibrosis (9).

Interleukin-1 (IL-1) plays a crucial role in mediating KD. Serum levels of IL-1β (10) and IL-1 related proteins are upregulated in the peripheral blood of KD patients during the acute phase of illness (11). IL-1 is required for pathogenesis in the *Lactobacillus casei* (LCWE) induced murine model of KD vasculitis (12–14), as evidenced by the observation that the IL-1 receptor antagonist anakinra can suppress disease in the model (12, 13). Recent studies also support an essential role for IL-1 in the *Candida albicans* model of KD (CAWS) (15–17).

The LCWE-induced murine model of KD vasculitis captures many of the features of human KD (18, 19). Like human KD, the murine model develops a much more vigorous disease phenotype in young animals and a greater severity in males. Importantly, the LCWE-induced KD murine model replicates most of the salient histopathological features of human KD (8, 18), such as the presence of intimal hyperplasia, adventitial and myocardial fibrosis, as well as elastin destruction and neo-elastic lamina formation (8). Additionally, many immunologic features and similarities of the disease are shared by both humans and mice (20, 21). The cytokine profile of the acute phase of the mouse model also bears strong similarity to that of the human disease, with elevations of IL-1β, TNF-α, IL-6, MCP-1, and IL-10 in the serum, as well as clinical symptoms of dysregulated temperature (20, 22). Human transcriptome, human genetic data and experimental murine models of KD all support a key role for IL-1β in the pathogenesis of KD, and also show the effectiveness of the LCWE model in terms of translational features to humans, such as histopathologic and even echocardiographic changes (10–17, 23–26).. Furthermore, the mouse model reliably predicts treatment efficacy, including of IVIG treatment (22).

We hypothesized that changes in cardiac protein expression after anakinra therapy of LCWE-injected mice would highlight disease- and treatment-relevant proteins. Here, we used a method of quantitative high-throughput sample preparation by magnetic-assisted digestion and tandem mass tag labelling (27) to analyze cardiac lysates from control and LCWE-injected mice developing KD vasculitis. By this method, we identified proteins expressed in the cardiac tissue including STAT3 that are responsive to anakinra treatment. However, despite increased STAT3 and IL-6 in the acute phase of disease, and the suppression of the acute phase reactant serum amyloid A (SAA) (28) by IL-6 blockade, their inhibition had no effect on the development of coronary and aortic vasculitis in the LCWE mouse model.

## Material and Methods

### Mice

WT C57BL/6 mice were obtained from the Jackson Laboratory and housed under specific pathogen-free (SPF) conditions in the American Association for Laboratory Animal Science (AALAS)-accredited facility at the Texas Biomedical Research Institute or Cedars Sinai Medical Center. All procedures involving animals were reviewed and approved by the institutional animal use and care committees (IACUC) of the Texas Biomedical Research Institute or Cedars Sinai Medical Center. Protocols were compliant with IACUC goals to reduce pain, suffering and distress. No adverse events occurred. Humane endpoints were euthanasia by IACUC approved methods.

### LCWE-Induced KD Vasculitis Mouse Model

*Lactobacillus casei* (ATCC 11578) cell wall extract (LCWE) was prepared as previously described (29). Male mice aged 5 weeks old were i.p. injected with 500 µg of LCWE or 500µl PBS. For anakinra treatment, anakinra was donated from unused patient samples (SOBI, Sweden) and injected i.p. at a dose of 25mg/kg/dose (approximately 500 µg per mouse) daily for five days, beginning one day before LCWE injection, as previously described (12). For Stattic experiments, Stattic (Selleckchem, Catalog No.S7024, 100µg in 50% DMSO : PBS solution) or vehicle control (50% DMSO : PBS solution) was administered by i.p. injection every three days starting 1 day prior to LCWE injection. Experiment was performed twice in two different laboratories, once with 13 male mice total, 6 in control group, and 7 in treated group; for the repeat, the experiment was performed with 10 male mice per group. For IL6R antagonism experiments, 600µg of anti-IL6R (MR-16-1, Genentech) or isotype control antibody were administered by i.p. injection every three days starting 1 day prior to LCWE injection. Experiments were performed with 13 male mice per group, with several repeat experiments. Sample sizes were based on number needed to see 50% differences at 80% power at a 0.05 significance level, given penetrance of Kawasaki model of 80%. All group assignments were randomized. No animals were excluded from analysis. Order of treatments, measurements and caging of animal was random to minimize confounders. All analysts (pathologists) and technicians were blinded. Mice were euthanized for tissue harvest at day 7 or 14 post LCWE (as indicated) and perfused with PBS. Hearts were removed and embedded in Optimal Cutting Temperature (OCT) compound for histological examination. Abdominal aortas were photographed, the abdominal aorta diameter (measured at 5 points) and abdominal aorta area was quantified with the software Image J. Serial cryosections (7µm) of heart tissue were stained with hematoxylin and eosin (H&E) and histopathological scoring of coronary arteritis, aortic root vasculitis, and myocarditis were performed blinded (12, 18). Images were obtained using a Biorevo BZ-X710 (Keyence) microscope.

### Proteomics Analysis

The upper portion of the heart (50%, including the aortic root) was placed directly into RIPA buffer (Milipore, Rockville MD) and then into liquid nitrogen for storage for subsequent homogenization. For isobaric TMT labeling, homogenized protein was pooled from representative specimens as reference material prior to sample preparation from individual specimens. Whole protein lysate was incubated and bound to C8 magnetic beads (BcMg, Bioclone, Inc.). The proteins were reduced with 10mM dithiothreitol (DTT) and alkylated with 50mM iodoacetamide. Overnight proteolysis was performed in a 1:25 trypsin-to-protein ratio at 37°C. Released tryptic peptides from digested proteins, including reference material described above, were normalized using the Pierce Quantitative Colorimetric Peptide Assay (Thermo Scientific) and then modified at the N-terminus and at lysine residues with tandem mass tag (TMT)-6plex isobaric labeling reagents (Thermo Scientific). Each individual specimen was encoded with one of the TMT_127-131_ reagents, while reference material was encoded with the TMT_126_ reagent. To normalize across all specimens, TMT-encoded lysates from individual specimens were mixed with the reference material in equal ratios. These TMT peptides were cleaned with C18 ZipTips (Milipore).

### Mass Spectrometry

The trypsin-digested TMT-labeled extracts were analyzed by nanoLC-MS consisting of an UltiMate 3000 Nano LC System and an LTQ-Orbitrap Elite mass spectrometer (Thermo Fisher). Reversed-phase liquid chromatography was performed using a 20 cm × 75 μm ID column packed with XBridgeTM BEH C18 media (2.5 μm, 130 Å). The flow rate was maintained at 200 nL/min. A full scan MS acquired in the range 300 ≤ m/z ≤ 2000 was followed by 10 data-dependent MS/MS events on the 10 most intense ions. The mass resolution was set at 60000 for full MS. The dynamic exclusion function was set as follows: repeat count, 1; repeat duration, 30 s; exclusion duration, 30s. HCD was performed using normalized collision energy of 35% and the activation time was set as 0.1 ms. Probability based protein database searching of MS/MS spectra against the Trembl protein database (release April 25, 2018; 114,759,640 entries) was performed with a 10-node MASCOT cluster (v.2.3.02, Matrix Science) with the following search criteria: 10 ppm precursor ion mass tolerance, 0.05 Da product ion mass tolerance, 3 missed cleavages, trypsin, carbamidomethyl cysteines as a static modification, oxidized methionines and deamidated asparagines as variable modification, an ion score threshold of 20 and TMT-6plex for quantification.

### Immunohistochemistry

Heart tissues were fixed in 10% neutral buffered formalin, processed conventionally, embedded in paraffin, cut at 5 microns transversely through the aortic root, H&E stained and scored by board-certified veterinary pathologists blinded to the grouping. Routine immunohistochemistry for STAT3 (Invitrogen, Thermo Fisher cat# MA1-13042, 1:1600 dilution) was performed on serial sections adjacent to the H&E sections. Briefly, slides were cut at 5 microns, dried (60°C for 1 hr), deparaffinized through 3 changes of xylene, 2 changes of absolute alcohol, 2 changes of 95% alcohol, and rinsed in distilled water. Antigen retrieval was conducted using Target Retrieval Solution pH 9 (Dako cat# S2367) in a decloaking chamber (Biocare Medical). Slides were allowed to cool, rinsed with Tris Buffered Saline with Tween (Dako cat# S3006) and treated with Dual Endogenous Enzyme block (Dako cat# S2003). Slides were incubated with anti-STAT3, as well as isotype control antibodies (Rabbit Isotype Control, Invitrogen, cat# 02-6102. 1:1600 dilution). Slides were then treated with ADVANCE HRP Link (Dako cat# K4069), ADVANCE HRP Enzyme (Dako cat# K4069), DAB substrate-chromagen (Dako cat# K3467), and Hematoxylin counterstain (Statlab cat# SL200), dehydrated, cleared, and cover-slipped. For scoring immunohistochemistry, a scale of 0 – 5 (Normal= 0, Minimal= 1, Mild= 2, Moderate= 3, Moderately Severe= 4, Severe= 5), was used for degree of positively stained cells in and around the coronary artery, aortic root, valve and coronary arteries.

### Immunofluorescence

OCT frozen 7μm heart tissue sections were fixed in acetone, washed in PBS, and stained overnight with 1 μg/ml of the following antibodies: Phospho-STAT3 (9145; Cell Signaling Technologies) or control Rabbit IgG (ab17180; Abcam). Sections were incubated with 2μg/ml Donkey Anti-Rabbit Alexa Fluor 555 (A-31572, Invitrogen). Nuclei were counterstained with Mounting Medium with DAPI (ab104139, Abcam). Images were obtained using a Biorevo BZ-9000 (Keyence) fluorescent microscope and mean fluorescence intensity (MFI) were quantified using Image J software.

### Serum Analysis

Quantification of IL-6 in serum collected 2 weeks post-LCWE injection in mice treated with or without anakinra was performed with a highly sensitive multiplex enzyme-linked immunosorbent assay (ELISA) using electrochemiluminescence detection technology (Meso Scale Discovery [MSD], Rockville, Maryland, USA). Briefly, serum was prepared according to MSD protocol and were measured in duplicate: 50µl of 2-fold diluted samples and calibrators were pipetted in each well and incubated for 2 hours at room temperature with shaking. Following washing the plates three times with wash buffer (1xPBS and 0.05% Tween20), 25µl of MSD detection antibody solution was added to each well and incubated for 2 hours at room temperature with shaking. Plates were washed three times with wash buffer and then 150µl of MSD Read Buffer was added to each well. The plates then were read and analyzed by MSD instrument (MESO Quicklex S 120). Serum SAA levels were determined by ELISA (mouse SAA Elisa kit, Tridelta Development Ltd.) according to the manufacturer’s instructions.

### Western Blot Analysis

Frozen mouse hearts (-80°C) were immersed into RIPA lysis buffer and homogenized using a Polytron. Protein content of whole lysate was quantified, and samples were prepared for western blot using 5x Laemmli buffer. The membranes were incubated with antibodies against STAT3 (1:1000, Cell Signaling Technology, #9139) and phospho(Y705)STAT3 (1:1000, Cell Signaling Technology, #9145). Protein content was normalized to GAPDH (1:1000, Cell Signaling Technology, #5174). Due to space limitation we show the cropped blots in the manuscript. The complete gels are included in the supplementary data.

### Statistics

For proteomics protein expression evaluation, Scaffold Q+ (Scaffold_4.6.2, Proteome Software Inc.) was used to quantitate Label Based Quantitation (TMT) peptide and protein identification. Differentially expressed proteins were determined by applying Mann-Whitney Test with unadjusted significance level p<0.05 corrected by Benjamini-Hochberg. Principal component analysis (PCA) was performed using XLSTAT Software (Addinsoft, Paris, France). Heat map construction and hierarchical clustering using one minus Pearson’s correlation was performed using Morpheus (http://software.broadinstitute.org/morpheus). Pathway analysis performed using STRING (30) (https://string-db.org/). All other data were analyzed by Student’s t test for single comparisons or one-way ANOVA with Tukey’s multiple testing correction for multiple comparisons. Differences between groups were considered to be significant at a P value of <0.05. Graphical representation of data and statistical analysis was performed with GraphPad Prism 5.0 (GraphPad Software, Inc., San Diego, CA). Receiver operator curve was constructed using STATA version 11 (STATA Corp, College Station, TX).

## Results

### The Myocardial Proteome in the LCWE-Induced KD Vasculitis Model Shows a Distinct Protein Expression Profile Which Is Partially Normalized by Anakinra Treatment

**Figure 1:**
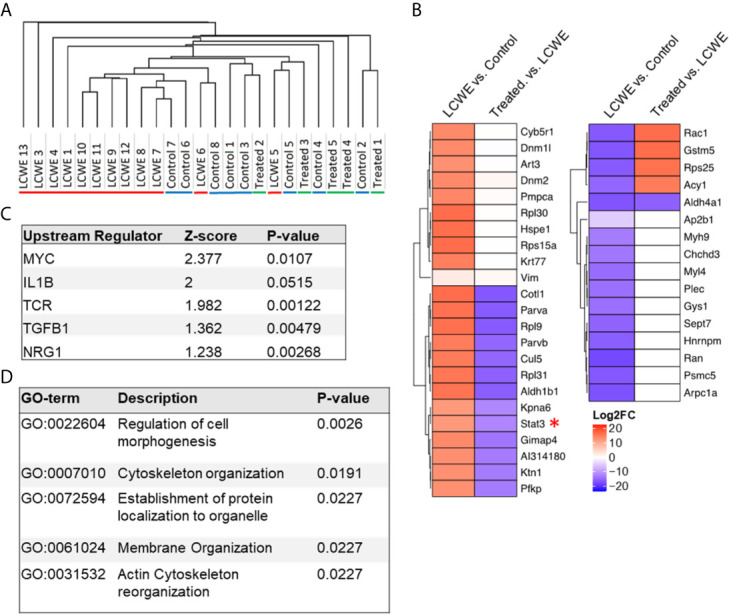
Proteomics analysis of cardiac tissue using the LCWE induced mouse model of KD. **(A)** Hierarchical one-minus Pearson’s clustering. “LCWE” indicates LCWE-injected mice (n=13), “Control” indicates PBS injected mice (n=8), and “Treated” indicates LCWE injected mice treated with anakinra (n=5). **(B)** Heatmap of fold change for proteins found differentially expressed (FC > 2, p<0.05) between LCWE-injected mice and PBS controls or between anakinra-treated LCWE and untreated LCWE mice. **(C)** Top regulators (ranked by z-score) identified by IPA Upstream Analyses of proteins differentially expressed (FC > 2, p<0.05) between KD mice and PBS controls. D) STRING Gene Ontology Biological Process analyses of proteins differentially expressed (FC > 2, p<0.05) between KD mice and PBS controls.

The LCWE model induces a profound inflammatory response in the tissue surrounding the aortic root and vasculitis of the coronary arteries which extends well into the mid portion of the mouse heart. We performed proteomics analysis to evaluate the changes in cardiac tissue protein expression during LCWE-induced KD vasculitis and the response to anakinra treatment. Five-week old male WT mice were injected with either PBS or LCWE, and a subset of LCWE-injected mice were treated with anakinra daily, for five days. Two weeks later, cardiac tissues were collected and analyzed as previously described (31). We visualized protein expression differences between the groups using Pearson’s hierarchical clustering (**Figure 1A**). LCWE-injected mice formed a distinct and minimally overlapping cluster from PBS controls. Two of the thirteen mice injected with LCWE clustered with the PBS controls, reflective of the incomplete penetrance of the LCWE-model (70-80% penetrance), as previously described (32) (**Figure 1A**). Furthermore, LCWE-injected mice treated with anakinra clustered with PBS control mice, indicating that IL-1Ra treatment suppressed the LCWE-induced proteomic signature (**Figure 1A**). Principal component analysis (PCA) also suggested the presence of three distinct and minimally overlapping groups, differentiating LCWE-injected KD mice, LCWE-injected mice treated with anakinra, and control mice (**Supplementary Figure 1**). Next, we performed differential protein expression analysis between the groups. We found 23 proteins were significantly upregulated (FC >2 and p-value <0.05) and 16 proteins were significantly downregulated (FC >2 and p-value <0.05) in cardiac tissue between LCWE-injected KD mice and PBS-injected controls (**Figure 1B**). Notably, expression of the transcription factor STAT3 was greatly increased in cardiac tissue of LCWE-injected mice. Approximately half of the proteins differentially regulated in LCWE-injected KD mice were normalized by anakinra treatment, including STAT3 (**Figure 1B**). Overall, these data indicate that LCWE induces changes in cardiac protein expression which are partially normalized by anakinra treatment.

The differentially expressed proteins (LCWE vs. PBS control) were then analyzed using IPA upstream regulator analysis (**Figure 1C**) and STRING database analysis of Gene Ontology (**Figure 1D**). IL-1β was amongst the top upstream regulators when ranked by Z-score, in line with previous investigations in this model (12–14, 19), and our findings that anakinra treatment suppressed the LCWE-induced proteome changes. STRING analysis identified pathways involved in cellular morphogenesis and cytoskeletal organization (**Figure 1D**; **Supplementary Table 1**). These pathways may be reflective of the signals driving the pronounced myofibroblast proliferation that occurs in cardiac tissues and especially around inflamed vascular tissue during KD (8).

### Expression of STAT3 Is Increased in Cardiac Tissue in the LCWE-Induced KD Mouse Model of Vasculitis

**Figure 2:**
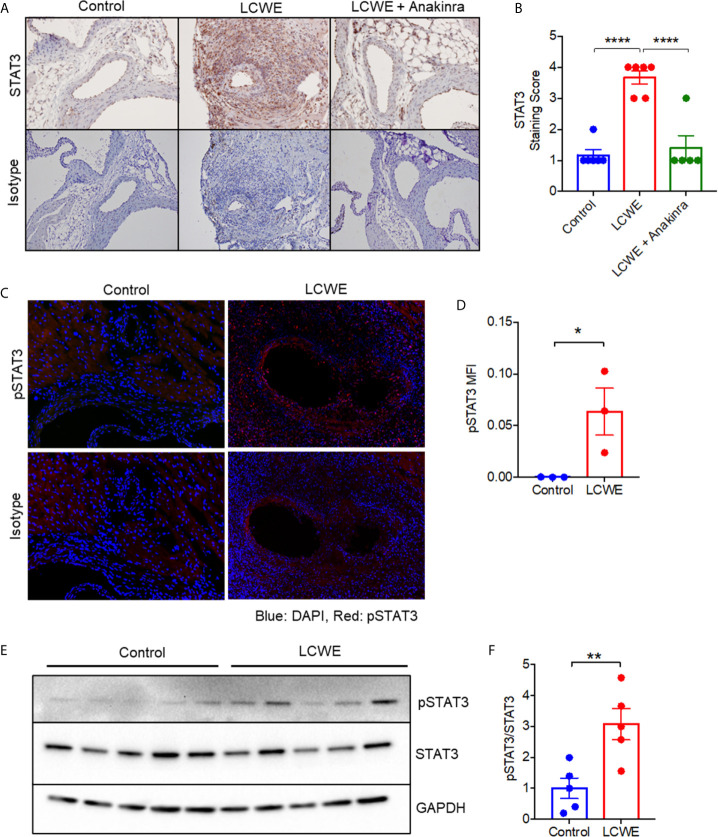
STAT3 expression in the LCWE-induced model of vasculitis and response to anakinra treatment. **(A)** Representative images of immunohistochemistry staining for STAT3 in murine heart of PBS control, LCWE injected or LCWE injected mice treated with anakinra. **(B)** STAT3 staining score in PBS control, LCWE injected and LCWE injected mice treated with anakinra. **(C)** Representative images of pSTAT3 immunofluorescence staining in murine heart tissue from PBS control and LCWE injected mice. **(D)** Quantification of pSTAT3 immunofluorescence staining in murine heart tissue. **(E)** Western blot of pSTAT3 and STAT3 in murine heart tissue from PBS control and LCWE injected mice. **(F)** Quantification of pSTAT3/STAT3 ratio from Western Blot. Data was analyzed by one-way ANOVA with Tukey’s multiple testing comparison **(B)** or Student’s t test **(D, F)**. * p < 0.05, ** p < 0.01, **** p < 0.0001.

Given the known roles of STAT3 in the immune response (33), we next sought to validate the upregulation of STAT3 in the LCWE model by immunohistochemistry. Five-week old WT mice were injected with either PBS, LCWE or LCWE in combination with anakinra treatment. Two weeks later, cardiac tissues were collected, and sections were stained with isotype control or anti-STAT3 antibody. STAT3 was significantly upregulated in the cardiac tissue of LCWE-injected mice (**Figures 2A, B** **)**, confirming our proteomics data. The majority of the increased STAT3 expression was observed in the peri-vascular and peri-aortic root inflammatory zones, and not in the myocardial tissue of LCWE-injected mice (**Figure 2A**). Importantly, treatment with anakinra normalized STAT3 staining (**Figures 2A, B** **)**.

STAT3 tyrosine phosphorylation (Y705) results in STAT3 activation and transcriptional activity. To determine if the STAT3 protein present in cardiac tissue of LCWE-injected mice was phosphorylated, we performed immunofluorescence staining for p(Y705)STAT3 on heart sections, and found significantly greater expression of pSTAT3 in LCWE-injected mice compared to PBS controls (**Figures 2C, D** **)**. Western blot analysis of whole heart lysates from PBS or LCWE injected mice confirmed an increased ratio of p(Y705)STAT3 to total STAT3 in cardiac tissue from LCWE injected mice (**Figures 2E, F** **)**, indicating STAT3 is activated in LCWE-injected mice.

### The STAT3 Activator, IL-6, Is Enhanced in LCWE-Injected Mice and Regulated by Anakinra

**Figure 3:**
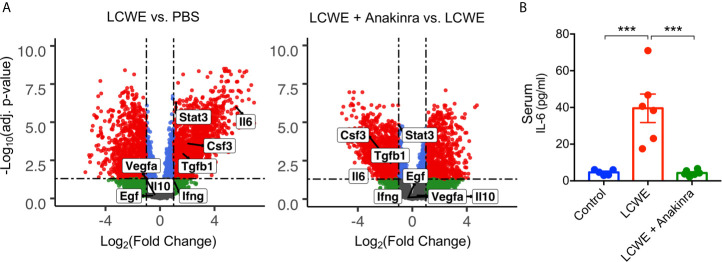
IL-6 expression in the LCWE-induced KD mouse model. **(A)** Volcano Plots demonstrating differential expression of *Stat3*, *Il6, Csf3, Tgfb1, Ifng, Il10, Vegfa and Egf* in the abdominal aorta of PBS vs. LCWE treated mice and LCWE vs. LCWE + anakinra treated mice. *Csf2* was not detected. Red represents FDR < 0.05 and FC > 2, blue represents FDR < 0.05 and FC < 2, green represents FDR > 0.05 and FC < 2. **(B)** Serum levels of IL-6 in PBS, LCWE and LCWE + anakinra treated mice. Data was analyzed by one-way ANOVA with Tukey’s multiple testing comparison **(B)**. ***p < 0.0001.

STAT3 can be activated by a range of cytokines and growth factors including IL-6, IFNγ, TNFα, CSF2, CSF3, IL-10, TGF-β, VEGF and EGF. To determine the possible upstream regulators of STAT3 in the LCWE-induced mouse model of KD vasculitis, we utilized our previously published RNA-seq data (GSE141072) (19) from the abdominal aorta of mice treated with PBS, LCWE or LCWE and anakinra. We analyzed this dataset for the expression of *Stat3* and the genes encoding the cytokines and growth factors listed above (**Figure 3A**). We found significantly (FDR < 0.05, FC > 2) enhanced expression of *Stat3*, *Il6*, *Csf3* and *Tgfb1* in abdominal aorta of mice treated with LCWE, and these were all downregulated with anakinra treatment (**Figure 3A**). Of the candidate STAT3 activators, the most highly upregulated was *Il6*. IL-6 is a well characterized inducer of STAT3 activation and expression, and high serum IL-6 levels are found in the acute phase of KD (34, 35). We therefore analyzed serum levels of IL-6 in this model. We found high levels of IL-6 in the serum of LCWE-injected mice which were reduced to control levels by anakinra treatment (**Figure 3B**). These data suggest STAT3 activation and phosphorylation may be regulated, in part, by IL-6 in the LCWE model.

### Treatment With the Small Molecule STAT3 inhibitor, Stattic, Fails to Attenuate LCWE-Induced KD Vasculitis Despite a Reduction of STAT3 Tyrosine Phosphorylation in Heart Tissue

**Figure 4:**
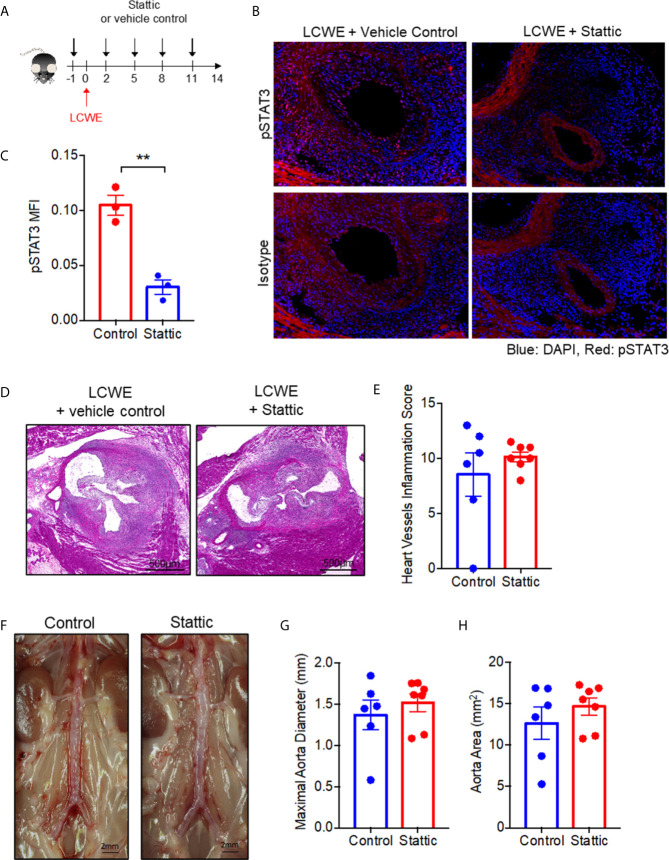
STAT3 inhibition fails to attenuate LCWE-induced KD vasculitis. **(A)** Schematic of the experimental design. **(B)** Representative images of pSTAT3 immunofluorescence staining in murine heart tissue from LCWE-injected mice treated with vehicle control of Stattic. **(C)** Quantification of pSTAT3 immunofluorescence staining in murine heart tissue. **(D, E)** Representative H&E stained heart sections **(D)** and heart vessel inflammation scores **(E)** of LCWE-injected WT mice treated with either vehicle control or the STAT3 inhibitor Stattic at 2 weeks post-LCWE injection. Scale bars, 500µm. **(F)** Pictures of the abdominal aorta of LCWE-injected WT mice treated with either vehicle control or Stattic at 2 weeks post-LCWE injection. **(G, H)** Maximal abdominal aorta diameter **(G)** and abdominal aorta area **(H)** of LCWE-injected WT mice treated with either vehicle control or Stattic at 2 weeks post-LCWE injection. Results are representative of two independent experiments (n = 6-7 mice per group). Data was analyzed by Student’s t test. ** p < 0.01.

Given STAT3 expression and phosphorylation was enhanced in the LCWE induced mouse model, we hypothesized that STAT3 may contribute to disease pathogenesis. To test the role of STAT3 in LCWE-induced KD vasculitis, we examined the impact of treatment with the small molecule STAT3 inhibitor, Stattic, on disease pathogenesis. LCWE-injected mice were treated with Stattic or vehicle control (**Figure 4A**). Immunofluorescence of heart sections confirmed that Stattic inhibited the LCWE-induction of STAT3 phosphorylation (**Figures 4B, C** **)**. However, Stattic treatment had no significant effect on heart vessel inflammation (**Figures 4D, E** **)** or AAA development (**Figures 4F–H**; **Supplementary Figure 3**). These results indicate that STAT3 does not play a pathogenic role in the acute phase of LCWE-induced KD vasculitis.

### Treatment With an IL6R Antagonist Fails to Attenuate LCWE-Induced KD Vasculitis Despite Suppression of Acute Phase Reactant, Serum Amyloid A

**Figure 5:**
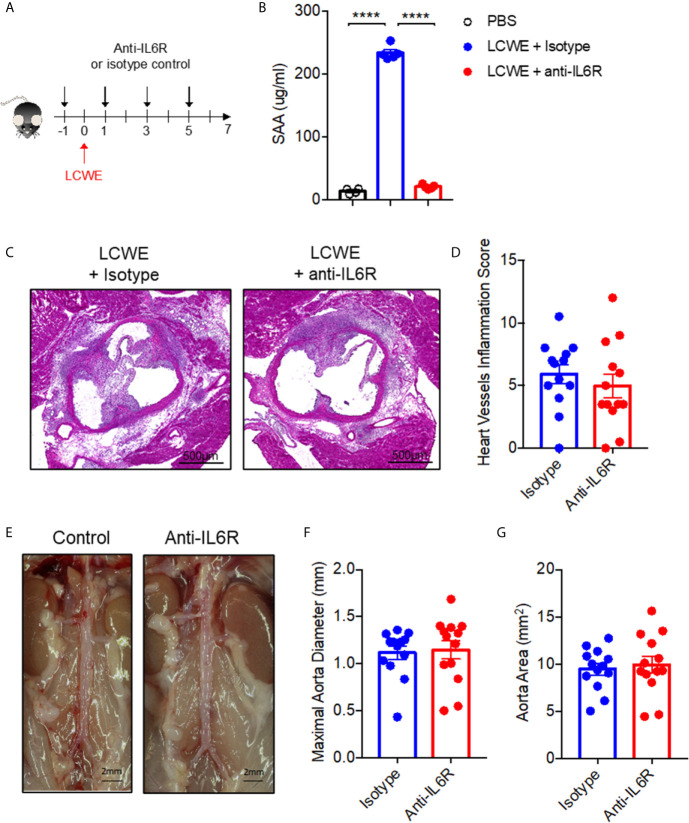
Blocking IL-6R signaling fails to attenuate LCWE-induced KD vasculitis. **(A)** Schematic of the experimental design. **(B)** SAA concentration in serum from PBS or LCWE mice treated with isotype control or anti-IL6R antibody. **(C, D)** Representative H&E stained heart sections **(C)** and heart vessel inflammation scores **(D)** of LCWE-injected WT mice treated with either isotype control or anti-IL6R antibody at 1-week post-LCWE injection. Scale bars, 500µm. **(E)** Pictures of the abdominal aorta area of LCWE-injected WT mice treated with either isotype control or anti-IL6R antibody at 1-week post-LCWE injection. **(F, G)** Maximal abdominal aorta diameter **(F)** and abdominal aorta area **(G)** of LCWE-injected WT mice treated with either isotype control or anti-IL6R antibody at 1-week post-LCWE injection. Results are from two independent experiments combined (n = 13 mice per group). Statistical analysis was performed by one-way ANOVA with Tukey’s multiple testing comparison **(B)** or Student’s t test **(D, F, G)**. ****p < 0.0001.

Next, to determine the role of IL-6 in LCWE-induced KD vasculitis, we examined the impact of treatment with an IL-6R antagonist antibody on disease pathogenesis. LCWE-injected mice were treated with anti-IL6R or isotype control (**Figure 5A**). Antibody treatment suppressed LCWE-induced serum amyloid A (SAA) expression is serum (**Figure 5B**). However, IL6R antagonism had no significant effect on heart vessel inflammation (**Figures 5C, D** **)** or AAA development (**Figures 5E, G**; **Supplementary Figure 4**). These results indicate that IL6 does not play a pathogenic role in the acute phase of the LCWE model, and that despite attenuation of acute phase reactant (SAA), IL-6 blockade does not rescue the focal vasculitis in this model.

## Discussion

In this study, we show that the myocardial proteome undergoes significant remodeling during LCWE-induced murine KD vasculitis, and that this expression pattern is partially normalized by anakinra blockade of the IL-1 receptor. We identified STAT3 and IL-6 as highly expressed in cardiac tissue of LCWE-injected mice, and normalized by anakinra. However, blockade of STAT3 or IL-6 did not alter acute disease in the LCWE-induced mouse model of KD vasculitis. Despite a decline in acute phase reactants, histologic evidence of vascular inflammation remained unchanged

Using proteomic analysis of myocardial tissue, we identified a set of proteins differentially expressed during LCWE-induced KD vasculitis in mice, which were partially normalized by anakinra treatment. STAT3 was amongst the most highly up-regulated proteins, and was suppressed by anakinra treatment. Furthermore, we found STAT3 tyrosine phosphorylation was enhanced in cardiac tissue from LCWE-injected mice compared to controls, indicating activation of the pathway. STAT3 is a transcription factor of major importance in both cardiac function and the immune system (36). STAT3 can be expressed by many different cell types and has a diverse set of functions including regulation of inflammation as well as cell growth, proliferation, differentiation, migration, and apoptosis. The multi-faceted role of STAT3, in particular its role both in regulation of inflammation and promotion of immune response, may be the reason that blockade of the IL-6 pathway did not result in resolution of local inflammation (33). STAT3 gain of function mutation is associated with autoimmune disease, potentially by impairing development of regulatory T cells and promoting activation of T helper type 17 cells (37). However, STAT3 signaling also plays a role in attenuating acute inflammatory responses in phagocytes and dendritic cells (38).

IL-6 is a pleiotropic inflammatory cytokine that can affect multiple cell types either through binding to the membrane bound IL-6 receptor (classical IL-6 signaling), which is believed to mediate the anti-inflammatory and regenerative activities, or through binding to soluble IL-6 receptors (IL-6 trans-signaling), which mediates the pro-inflammatory responses (39, 40). IL-6 participates in the pathogenesis of several inflammatory diseases, and inhibitors of IL-6 are commonly used to treat rheumatoid arthritis and other classical inflammatory diseases such as Crohn’s disease and psoriasis (40). IL-6 is also a potent inducer of STAT3 activation and expression. Like STAT3, we found that IL-6 was enhanced in the LCWE model of KD vasculitis and its expression was reduced with anakinra treatment. This is in line with KD patient data, which shows IL-6 is expressed during the acute phase of disease and is decreased during convalescence (34, 35). IL-6 plays a pathogenic role in a number of inflammatory and auto-immune diseases. Tocilizumab, a recombinant humanized monoclonal antibody that targets the soluble and membrane-bound IL-6 receptor to inhibit IL-6 signaling, is used for the treatment of various inflammatory diseases and more recently COVID-19 and MIS-C (41, 42). The high serum IL-6 levels during the acute phase of KD led to the hypothesis that tocilizumab may be therapeutically beneficial for the treatment of KD. However, similar elevations in IL-6 are also observed in febrile controls (34) and there appeared to be no correlation of serum IL-6 with development of coronary aneurysm or dilatation (35). In a small trial involving four IVIG-resistant patients, 2 developed giant coronary artery aneurysms following tocilizumab treatment despite reduced fever and CRP levels (43). This study indicated that IL-6R antagonism had no beneficial effect for the treatment of KD, however lacked statistical power to conclude a worsening of disease may occur. Using the LCWE-induced mouse model of KD vasculitis, we show that despite high IL-6 and STAT3 expression, suppression of these factors does not reduce the development of the cardiovascular lesions. The complex role of IL-6 and STAT3 in both inflammation and autoimmunity could explain the lack of effect.

IL-1β activates transcription factors that regulate IL-6 production (44). Indeed, IL-1β, the target of anakinra or canakinumab, strongly induces IL-6 production by several cell types, including vascular endothelial and smooth muscle cells (45, 46). We therefore propose that in the context of KD, IL-6 may be a bystander cytokine induced by IL-1β signaling, but plays no inflammatory role in the pathogenesis of cardiovascular lesion formation in the disease. Notably, we did observe that IL-6 inhibition resulted in reduced levels of SAA. SAA is a robust, clinically utilized acute phase reactant which is arguably as or more sensitive for infection and inflammation as the C-reactive protein, and plays active roles in innate immunity (28). The inhibition of SAA by tocilizumab with clear failure to inhibit vascular inflammation is of clinical importance. While measurement of acute phase reactants are one tool for diagnosis of rheumatic diseases including vasculitis (47), it has been recognized in the rheumatology literature that declines in acute phase reactants may not correlate with resolution of direct vascular injury in vasculitis (47, 48). Tocilizumab has been studied as a therapeutic agent in several vasculitis syndromes, including giant cell arteritis (49) (GCA) and Takayasu arteritis (50). While tocilizumab showed efficacy for GCA (49), it showed a trend of improvement but did not meet the study endpoint for Takayasu arteritis despite improvement in acute phase reactants (50). Recently, case reports of progression of vasculitis in patients with Takayasu arteritis despite treatment with tocilizumab have been published (51, 52). Our finding of failure of IL-6 blockade in the LCWE-model, along with lack of success in studies in human KD and potentially Takayasu arteritis, may suggest that more study is warranted for the use of tocilizumab in certain vasculitides.

Of the other proteins that were upregulated in myocardial tissue in the LCWE-induced KD murine model in our studies, a number appeared to be related to changes in cell morphogenesis. We posit that these proteins are reflective of the signals driving the pronounced myofibroblast proliferation that occurs in cardiac tissues and especially around inflamed vascular tissue during KD (8). Vimentin, a marker of mesenchymal transition (53), plays an active role in regulation of protein trafficking and gene expression, and has recently been described as being directly involved in angiogenesis *via* regulation of the Notch pathway (54). Other proteins that were upregulated in our study include Parva, Parvb, and Dnm2, which are also involved in cell morphogenesis (55). A second set of upregulated proteins of interest included Dnm1l and Dnm2. These proteins are involved in regulation of calcium signaling and mitochondrial fusion in cardiomyocytes and the development of cardiac hypertrophy (56, 57).

Myofibroblasts play a key role in the pathogenesis of remodeling and fibrosis in KD by proliferating and secreting matrix products that obstruct the coronary lumen (8). STAT3 has recently been identified as a putative mediator of dermal fibrosis in patients with scleroderma and its murine models, acting downstream of TGF-β to drive pro-fibrotic fibroblast responses (58, 59). Interestingly, the TGF-β signaling pathway has been linked to KD pathogenesis and is speculated to play a role in the generation of myofibroblasts in the disease (2, 9). Indeed, our data show TGF-β is an upstream regulator of proteins differentially expressed in cardiac tissue of LCWE-injected mice. Furthermore, we found *Tgfb* gene expression is enhanced with LCWE in abdominal aorta tissue and suppressed by anakinra. While we did not find a role for STAT3 in the acute phase of KD, it is possible that it does play a role in mediating fibrosis. Long-term studies examining fibrotic pathways are needed to investigate this possibility.

Limitations in the study are the inherent differences in murine vs. human biology; however, here the mouse model offers the most robust method to investigate details of pathology not typically available with human disease, and the LCWE model, as discussed, has played an important role in understanding pathophysiology of KD. Bias in the study was attenuated by the blinding of direct analysts such as pathologic interpretation of images, and of technicians during assays, as well as primary investigators, but cannot be completely eliminated.

## Data Availability Statement

The datasets presented in this study can be found in online repositories. The names of the repository/repositories and accession number(s) can be found below: ProteomeXchange, PXD024310.

## Ethics Statement

The animal study was reviewed and approved by Institutional animal use and care committees (IACUC) of the Texas Biomedical Research Institute and Cedars Sinai Medical Center.

## Author Contributions

MG, MA, RP, CCH, and TF conceived, designed, acquired and interpreted the data, drafted the manuscript ED, SK, RE, AG, and SM-I made substantial contributions to the acquisition and interpretation of data RP, MNR, JP, MG, MA, and TF made substantial contributions to the design of the work. All authors contributed to the article and approved the submitted version.

## Funding

1) The work of MG was supported by The Max and Minnie Tomerlin Voelcker Young Investigator Award and The San Antonio Medical Foundation 2018 Medical Research Award. 2) The work of RP was supported by the American Heart Association Career Development Award. 3) The work of MA was supported by the NIH Grant R01 AI072726. 4) The work of MR was supported by the NIH grant R01 HL139766.

## Conflict of Interest

The authors declare that the research was conducted in the absence of any commercial or financial relationships that could be construed as a potential conflict of interest.

## Supplementary Material

Supplementary Figure 1 Principal component analysis of proteomics data. Proteomics data clustering based on two primary factors, with the majority of mice in each group forming clusters as shown in circles.

Supplementary Figure 2 Western Blot data. Full western blot data from **Figures 2E, F**.

Supplementary Figure 3 Whole photographs of images used on **Figure 4F**.

Supplementary Figure 4 Whole photographs of images used on **Figure 5E**.

## Abbreviations

KD, Kawasaki Disease; LCWE, *lactobacillus casei cell wall extract;* MS, mass spectroscopy; IHC, immunohistochemistry; PBS, phosphate buffered saline; ELISA, enzyme linked immunosorbent assay.
