# Supplementary figures and images for: Inhibition of IL-6 in the LCWE Mouse Model of Kawasaki Disease Inhibits Acute Phase Reactant Serum Amyloid A but Fails to Attenuate Vasculitis
Source: Front Immunol. 2021 Apr 9;12:630196. doi: 10.3389/fimmu.2021.630196 (PMC8064710; doi:10.3389/fimmu.2021.630196)

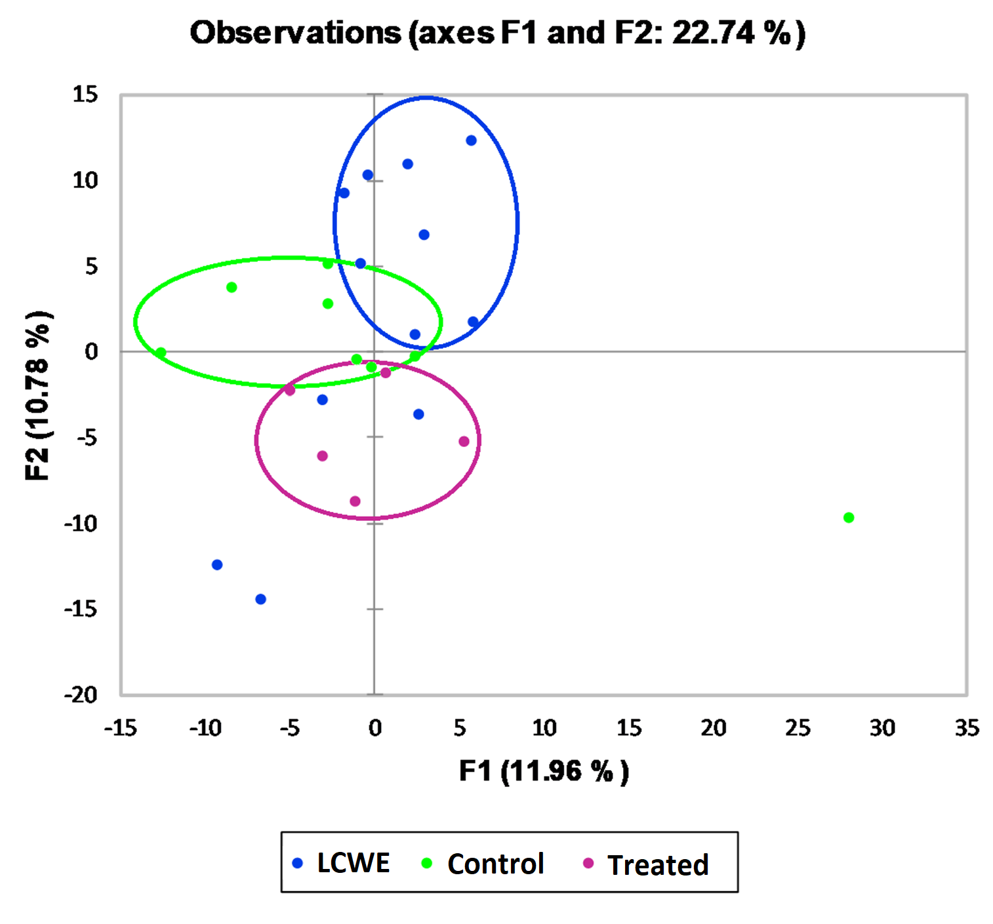

Supplement: Supplementary Figure 1 — Principal component analysis of proteomics data. Proteomics data clustering based on two primary factors, with the majority of mice in each group forming clusters as shown in circles. [file Image_1.tif]

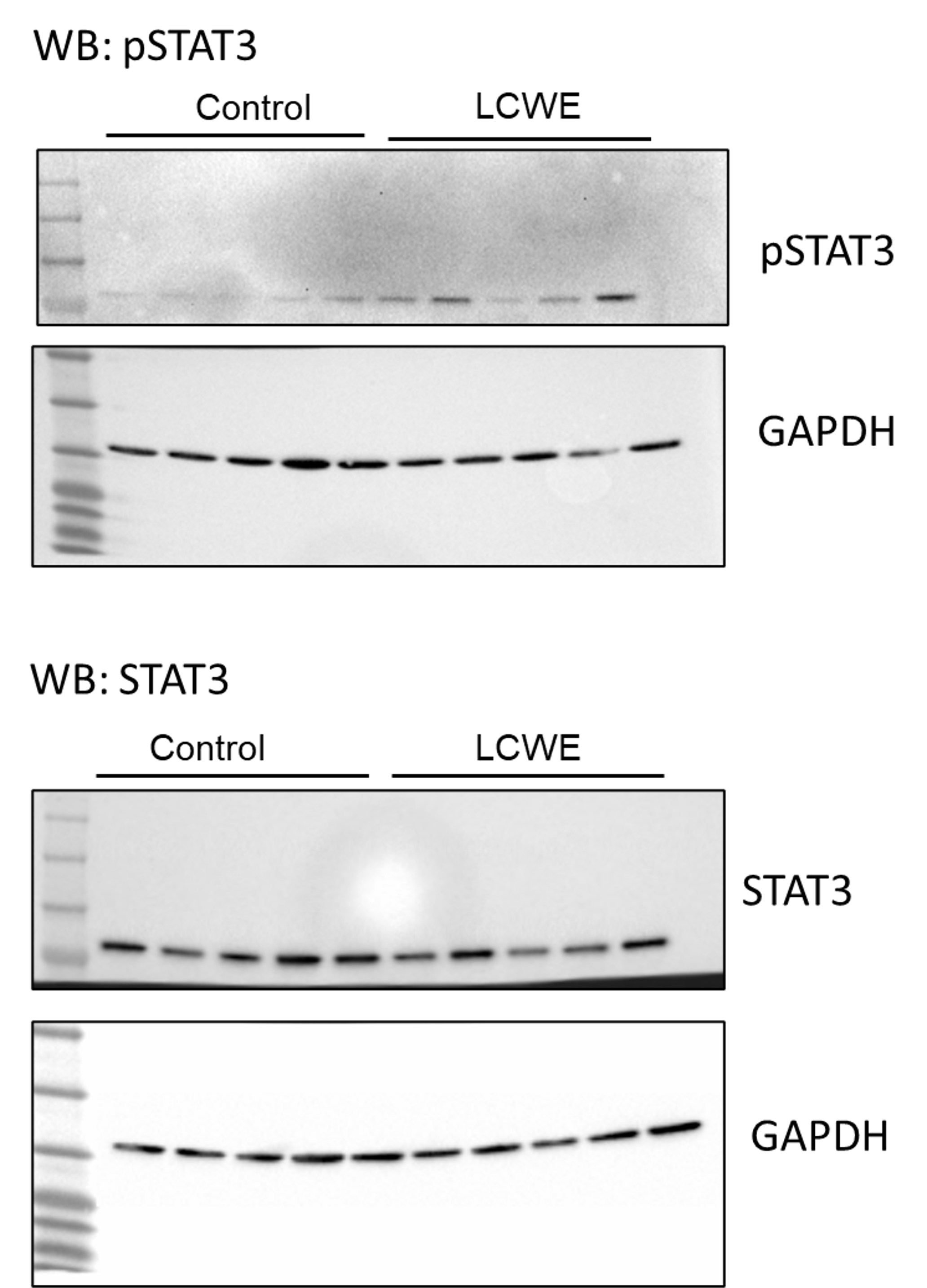

Supplement: Supplementary Figure 2 — Western Blot data. Full western blot data from Figures 2E, F . [file Image_2.tif]

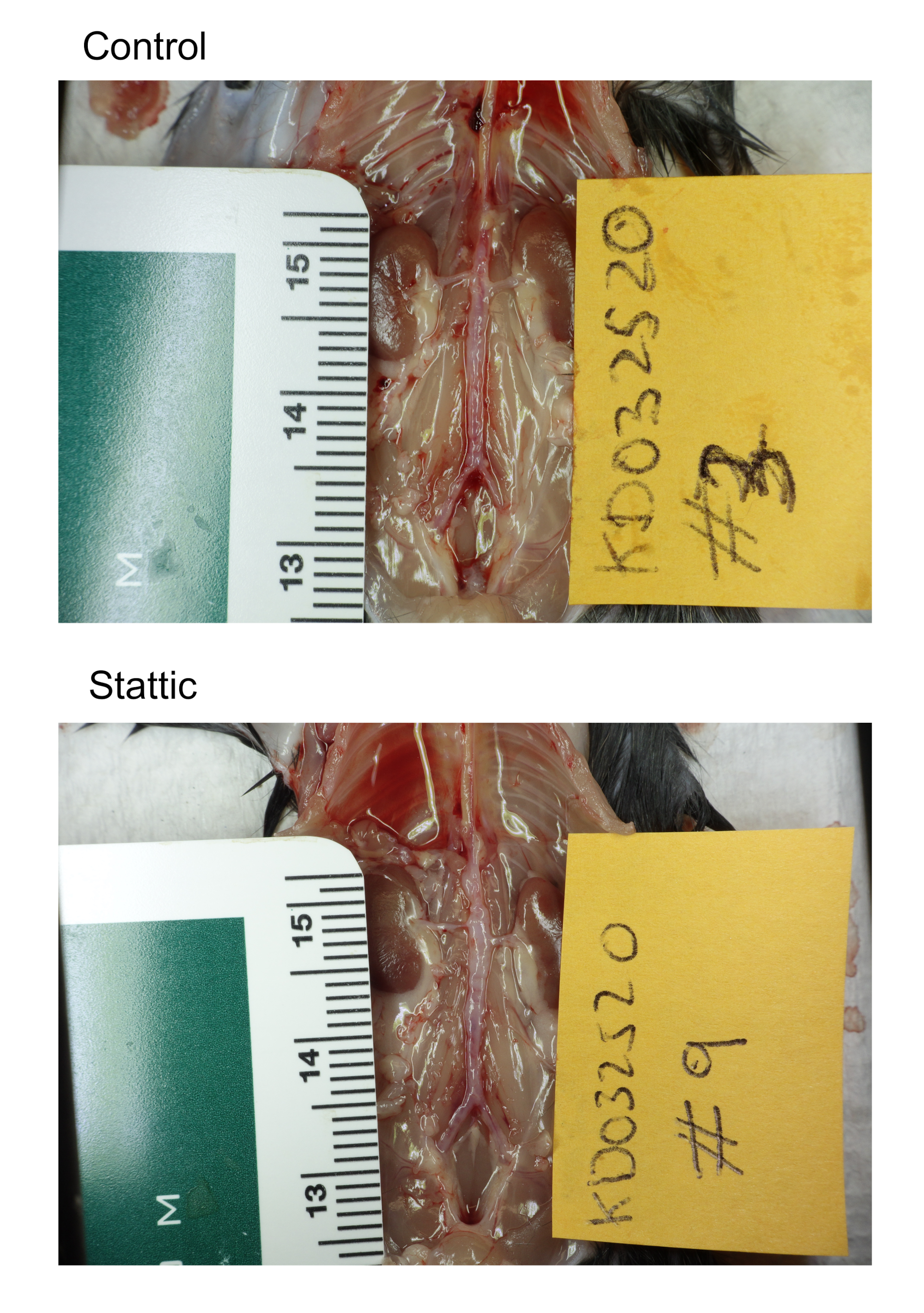

Supplement: Supplementary Figure 3 — Whole photographs of images used on Figure 4F . [file Image_3.tif]

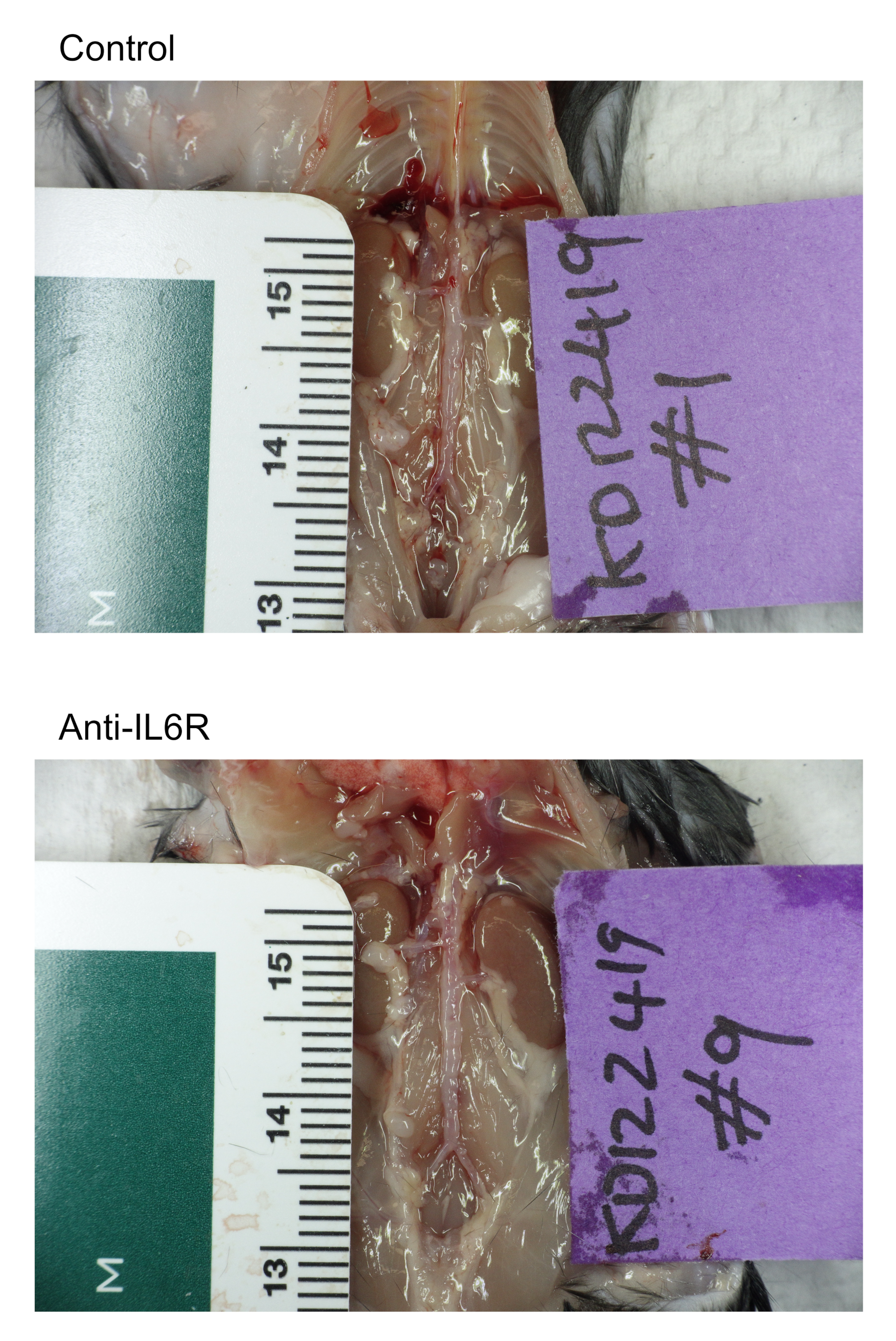

Supplement: Supplementary Figure 4 — Whole photographs of images used on Figure 5E . [file Image_4.tif]
