# Supplementary material for: Inhibition of IL-6 in the LCWE Mouse Model of Kawasaki Disease Inhibits Acute Phase Reactant Serum Amyloid A but Fails to Attenuate Vasculitis
Source: Front Immunol. 2021 Apr 9;12:630196. doi: 10.3389/fimmu.2021.630196 (PMC8064710; doi:10.3389/fimmu.2021.630196)
Supplement: Supplementary file 5 [file Table_1.docx]

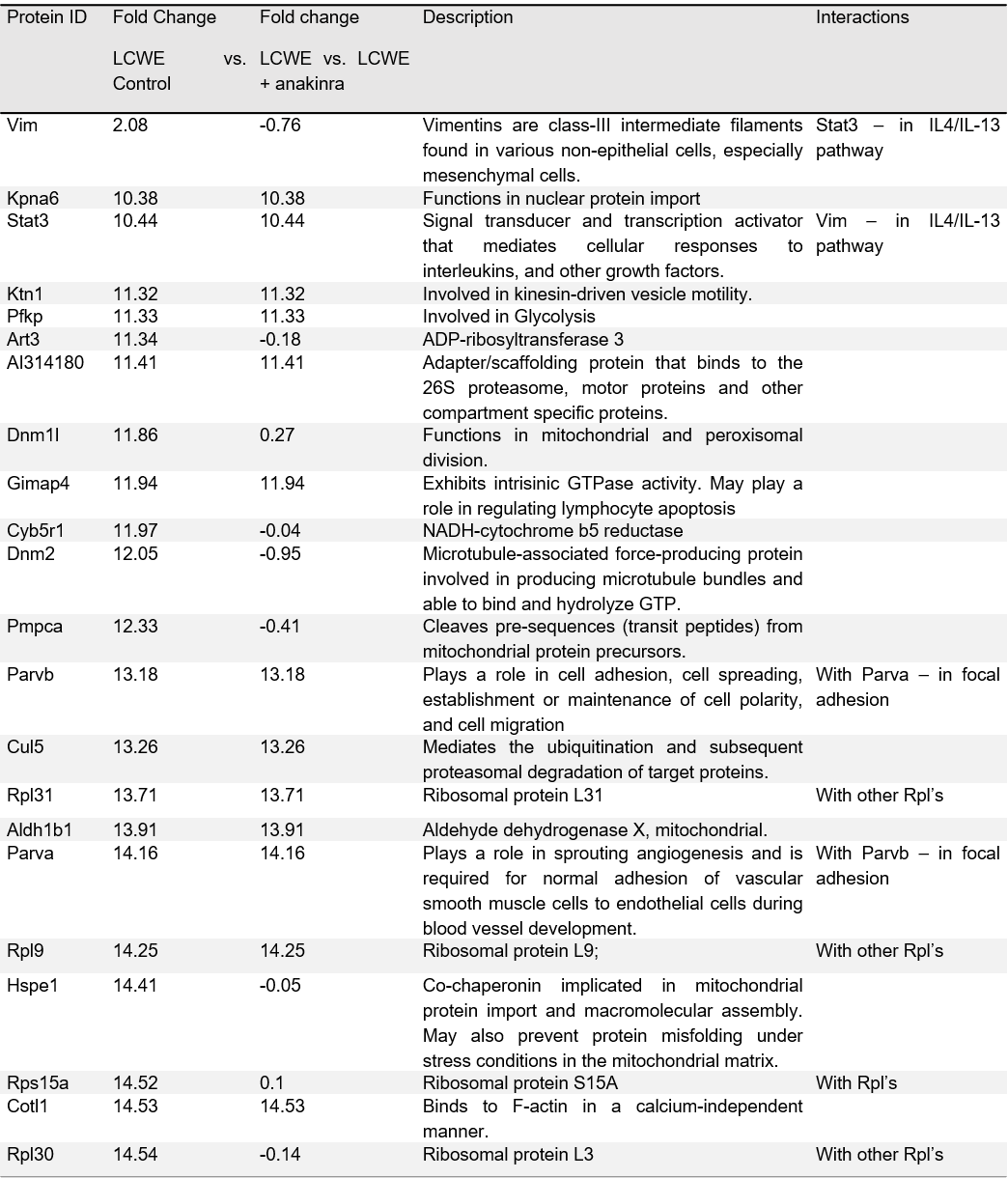


**Supplementary Table 1.** Proteins up regulated in LCWE injected mice as compared to saline injected controls.
